# Age-specific kinetics of neutralizing antibodies and infection enhancement among ≤1 year-old Indian infants

**DOI:** 10.3389/fcimb.2025.1538188

**Published:** 2025-02-27

**Authors:** Shweta Chelluboina, Akhilesh Chandra Mishra, Vidya Avinash Arankalle, Shubham Shrivastava

**Affiliations:** Translational Virology, Interactive Research School for Health Affairs (IRSHA), Bharati Vidyapeeth (Deemed to be University), Pune, India

**Keywords:** dengue, neutralizing antibodies, infection enhancement, infants, ADE

## Abstract

**Background:**

Infants born to dengue-immune mothers acquire maternal antibodies to dengue. Maternal antibodies decline over time, making infants susceptible to primary dengue infections. Another important concern is the role of maternal antibodies in causing antibody-dependent enhancement (ADE) during primary infections. In this study, we aimed to investigate the kinetics of dengue virus (DENV)-neutralizing antibodies and infection-enhancing activity in Indian infants.

**Methods:**

Healthy infants at birth (cord blood), and at 3, 6, 9, and 12 months of age (n=32/group) were included in this cross-sectional study. Serum samples were tested for neutralizing antibodies using the foci-reduction neutralization test and enhancing antibodies using the ADE assay against DENV1-4 serotypes.

**Results:**

Neutralizing antibody positivity declined with the increasing age of the infants. Undetectable levels of neutralizing antibodies to DENV1-4 serotypes were reported in 84% of infants by 9 months. Significantly lower neutralizing antibody titers were also reported in 9-month-old infants compared to that in 6-month-old infants and infants at birth. Comparable levels of enhancement of DENV1-4 infection at a particular dilution to at least one serotype were noted in infants at 3 and 6 months of age. Fold enhancement of DENV1-4 infection was found to be highest in 6-month-old infants at a dilution of 1:20. In summary, our data suggests that DENV infection–enhancing activity aligns with the decline of neutralizing antibodies.

**Conclusion:**

Our study indicates that maternally acquired neutralizing antibodies could be protective until 6 months of age and capable of facilitating ADE on exposure to dengue infections in later months of life.

## 1 Introduction

The incidence of dengue has grown drastically around the world with over 6.5 million cases and more than 7,500 deaths in 2023. The disease is endemic in more than 100 countries and is rapidly spreading to new areas in Europe, Eastern Mediterranean, and South America [Dengue - Global situation (World Health Organization, 2024) https://www.who.int/emergencies/disease-outbreak-news/item/2023-DON498]. India is a dengue-endemic country and as per the National Center for Vector-borne Diseases Control program, India reported 289,235 laboratory-confirmed dengue cases in 2023 [Dengue situation in India (National Center for Vector Borne Diseases Control, 2024) https://ncvbdc.mohfw.gov.in/index4.php?lang=1&level=0&linkid=431&lid=3715]. Dengue infection is caused by any one of the four serotypes of dengue virus (DENV). As the four serotypes are co-circulating, multiple exposures to different serotypes of DENV lead to heterologous dengue infection (Guzman et al., 2016).

Dengue is known to infect individuals of all age groups, but the highest incidences were commonly reported among young children, particularly infants (Kabra et al., 1999; Jain and Chaturvedi, 2010). Infants were at high risk of developing severe dengue illnesses. In Thailand, Vietnam, Myanmar, and Indonesia, approximately 5% of infants developed Dengue Hemorrhagic Fever (DHF)/Dengue Shock Syndrome (DSS) during 1996-1998 (Halstead et al., 2002). In India, infants comprised 20% of total DENV infections in an outbreak in Chennai (Kabilan et al., 2003). Maternal antibodies are usually known to protect infants from diseases. However, with the time-dependent decline in these antibodies, such protection is lost, making the infants susceptible to infections. As maternal antibodies are also known to interfere with vaccination in young infants, a careful balance between protection and interference is estimated to protect infants globally from childhood infections. In dengue, maternally acquired antibodies predispose infants to antibody-dependent enhancement (ADE) and severe clinical outcomes (Kliks et al., 1988; Halstead et al., 2002; Simmons et al., 2007). Infants under 1 year of age have experienced severe primary dengue infections in dengue-endemic regions such as Thailand and Vietnam (Watanaveeradej et al., 2003; Chau et al., 2009; Pengsaa et al., 2011; Guignard et al., 2019). Although initially protective, maternally acquired dengue antibodies decline to sub-neutralizing levels, potentially leading to antibody-associated infection enhancement with fatal outcomes in infants (Chau et al., 2008; Libraty et al., 2009; Castanha et al., 2016). A recent vaccine efficacy study observed the waning of antibodies among children, indicating a potential need to revise vaccination recommendations for this age group (Borja-Tabora et al., 2024). Given the increased risk of severe infection among infants, it becomes crucial to characterize the maternal and infant DENV immune profiles over time.

In our study, we sought to illustrate the enhancement of dengue infection among Indian infants. We examined different age groups in an infant cohort for the presence of acquired dengue antibodies by measuring indirect IgG levels. We also determined the kinetics of DENV-specific neutralizing antibodies and enhancement of dengue virus infection among all four dengue serotypes.

## 2 Methods

### 2.1 Cells and viruses

Vero (CCL-81, African green monkey kidney epithelial cells, ATCC, USA) were grown in minimal essential media (MEM) (Invitrogen, Carlsbad, CA, USA), supplemented with 10% v/v heat-inactivated fetal bovine serum (FBS, Life Technologies, CA, USA) and 1% penicillin-streptomycin (P/S) (Invitrogen, Carlsbad, CA, USA). K562 (human erythroleukemic cell line, NCCS, Pune) were cultured in 10% FBS containing RPMI 1640 medium (Sigma-Aldrich, St. Louis, MO, USA). Dengue virus strains used in this study include DENV-1 (S19, accession number MW191722), DENV-2 (S15, accession number MW191699), DENV-3 (S111, accession number MW192820) and DENV-4 (1024, accession number MG272272). The viruses were isolated in Pune, India from patients’ serum samples during the dengue season in 2016 (Mishra et al., 2018).

### 2.2 Samples

A cross-sectional set of 160 serum samples from healthy infants with no evidence of DENV infection was selected for this study. Samples collected from infants at birth (cord blood), and at 3, 6, 9, and 12 months of age were examined for neutralizing antibodies and ADE response. The cord blood and blood samples from infants were collected previously for different studies between 2016 to 2018 at Bharati Vidyapeeth Medical College & Hospital, Katraj, Pune (Malshe et al., 2019; Arankalle et al., 2019). An equal number of samples (n=32) were used for all the age groups.

### 2.3 Foci reduction microneutralization test

Conventional Vero-cell-based foci reduction microneutralization tests were performed. Foci reduction microneutralization test (FRNT) methodology as described by Mishra et al. (2018) was adapted to a 96-well plate format. Thus, 1 day before infection, Vero cells were seeded in a 96-well flat bottom plate at a density of 10,000 cells/well/100µl and incubated at 37°C and 5% CO_2_. In a 96-well plate, 60µl of serial 2-fold dilutions of serum samples (1:10 to 1:5120) were prepared in 2% MEM media and mixed with 60µl dengue viruses, yielding 25-60 foci per well and incubated for 1 hour at 37°C and 5% CO_2_. Furthermore, 50μl of the resultant serum-virus mixtures was added in duplicate wells onto pre-seeded plates and further incubated for 1.5 hours for DENV-2/4 and 2 hours for DENV-1/3. A semi-solid 1% overlay media prepared using carboxymethyl cellulose and 2% MEM was then added to the cells and further incubated for 2 days.

#### 2.3.1 Immunostaining

After 2 days post-infection, the overlay media was removed from the plates and the cell monolayer was fixed with 3.7% formaldehyde solution for 30 min. Cells were washed three times with PBS followed by permeabilization with 0.2% Triton X-100 in PBS for 10 min. Cells were washed thrice with PBST (0.02% Tween-20 in PBS) and incubated with 2.5% skim milk powder (MP Biomedicals, USA) for 1 hr. After washing with PBST, the cells were further incubated with 1:500 diluted HB112 primary antibody for 2 hours. Post-washing, the cells were then incubated with 1:1500 diluted secondary goat anti-mouse IgG HRP antibodies for 1 hour. After washing twice with PBST and three times with PBS, cells were stained with True Blue Peroxidase substrate (KPL, Sera Care, MA, USA) and incubated in the dark for 30 min. Virus-infected cells that appeared as blue foci were counted using the CTL Immunospot analyzer (Cellular Technology Ltd., USA).

#### 2.3.2 Neutralization titer calculation

The neutralization titer (FRNT_50_) of the test serum sample was defined as the reciprocal of the highest serum dilution at which the number of foci was reduced by 50% when compared with the average foci count of input virus control. The lower limit of quantitation (LLOQ) was 16, 13, 11, and 11 for DENV-1, DENV-2, DENV-3, and DENV-4 respectively. FRNT_50_ titer ≥ LLOQ to at least one dengue serotype was considered seropositive.

### 2.4 Antibody-dependent enhancement assay

For the ADE assay, 60µl of serial 10-fold dilutions of serum samples (1:10 to 1:100000) were prepared in 2% RPMI media and mixed with 60µl of DENV1-4 at the multiplicity of infection (MOI) 0.2 and incubated at 37°C and 5% CO_2_ for 1 hour. Furthermore, 50µl of the resultant mixture was added in duplicate wells onto seeded 96-well U bottom plates containing 20,000 K562 cells per well and further incubated for 2 hours. Cells were then washed twice with serum-free media and resuspended in 100µl of 2% RPMI and further incubated at 37°C and 5% CO_2_ for 24 hours. After 24 hours, the infected 96-well U bottom plates were centrifuged at 2,000 rpm for 5 mins and the supernatants were harvested and transferred to 96-well flat bottom plates. These supernatants were subjected to plaque assay on Vero cells to measure enhanced infection by counting infectious particles.

#### 2.4.1 Plaque assay

After being diluted in 2% MEM media, 50µl of neat supernatant and serially diluted supernatants were added onto pre-seeded Vero cell plates. The plates were incubated for 2 hours for virus adsorption at 37°C with a 5% CO_2_ incubator. After adsorption, 1% overlay media was added to each well and further incubated for 2 days at 37˚C in a 5% CO_2_ incubator. Infected cells were stained as described previously and counted using a CTL Immunospot analyzer (Cellular Technology Ltd., USA).

#### 2.4.2 Enhancement of infection calculation

The fold enhancement of infection was determined using the following ratio: (mean foci count at different sample dilutions)/(mean foci count in the absence of sample, no antibody control). The cut-off values for each serotype above which positive enhancement was seen at different dilutions were as follows: DENV1 ≥ 80, DENV-2 ≥ 20, DENV3 ≥ 100, and DENV4 ≥ 60 ffu/ml. These were calculated from the mean+3 SD values from dengue-naïve control samples.

### 2.5 Statistical analysis

All statistical analyses and graphical representations were performed using GraphPad Prism software version 10 (GraphPad Software Inc, San Diego, USA). The Mann–Whitney U-test was used to compare neutralization titers and fold enhancement values between different age groups. Spearman’s test was performed to analyze the correlation between enhancing dilution (dilution at which enhancement of infection was observed) and neutralizing titers for all the samples. One-way ANOVA analysis was performed to compare the mean enhancing virus titers of serum samples of infants across different age groups for each serotype. Tukey’s test was applied for multiple comparisons, and an adjusted P-value was reported for each comparison.

## 3 Results

A total of 160 serum samples including 51 IgG-anti-DENV positives and 109 IgG-anti-DENV negatives were tested for neutralizing antibody response using the FRNT and enhancing antibody response using ADE assay. Of the IgG-positive infants, 96% (49/51) had neutralizing antibodies and all of them exhibited enhancing antibody activity against more than one DENV serotype (**Table 1**). Of the 109 IgG-negative infants, 32 (29%) had neutralizing antibodies. This suggests that ELISA has less sensitivity when FRNT is considered the gold standard. Interestingly, 60%, 58%, and 52% of IgG-negative infants at birth, 9 months, and 12 months, respectively, did not possess either neutralizing or enhancing antibodies indicative of either an absence of DENV exposure or a lack of cross-reactive flavivirus antibodies. On the contrary, 69% (9/13) and 81% (17/21) of IgG-negative infants at 3 months, and 6 months, respectively, showed enhancing antibody activity suggestive of circulation of cross-reactive flavivirus antibodies leading to antibody enhancement in 3-month-old and 6-month-old infants.

**Table 1:** Relationship between anti-DENV-IgG, neutralizing, and enhancing antibodies in infants across different age groups.

Age group (each n=32)	Anti-DENV-IgG status and number	FRNT-, ADE- (%)	FRNT+ ADE- (%)	FRNT-, ADE+ (%)	FRNT+ ADE+ (%)	Total ADE+ (%)
Cord blood	Pos, 17	0 (0)	0 (0)	0 (0)	17 (100)	17 (100)
	Neg, 15	9 (60)	2 (13)	2 (13)	2 (13)	4 (27)
3 months	Pos, 19	0 (0)	0 (0)	1 (5)	18 (95)	19 (100)
	Neg, 13	1 (8)	3 (23)	2 (15)	7 (54)	9 (69)
6 months	Pos, 11	1 (9)	0 (0)	0 (0)	10 (91)	10 (91)
	Neg, 21	4 (19)	0 (0)	11 (52)	6 (29)	17 (81)
9 months	Pos, 1	0 (0)	0 (0)	0 (0)	1 (100)	1(100)
	Neg, 31	18 (58)	0 (0)	9 (29)	4 (13)	13 (42)
12 months	Pos, 3	0 (0)	0 (0)	0 (0)	3 (100)	3(100)
	Neg, 29	15 (52)	7 (24)	6 (21)	1 (3)	7 (33)
Total	Pos, 51	1 (2)	0 (0)	1 (2)	49 (96)$	50 (98)
	Neg, 109	47 (43)	12 (11)	30 (28)*	20 (18)#	50 (46)
**Grand total**	**160**	**48 (30)**	**12 (8)**	**31 (19)**	**69 (43)**	**100**

*14 monotypic (five for DENV-2, three for DENV-3, and six for DENV-4); **#**6 monotypic (four for DENV-2, one for DENV-3, and one for DENV-4); **$**multitypic antibody enhancing activity. Bold values indicate total number of cases n (%).

### 3.1 Maternally transferred neutralizing antibodies wane rapidly among infants during the early months of life

Maternally transferred neutralizing antibodies to at least one serotype were detected in 66% and 88% of infants at birth and 3 months, respectively. Neutralizing antibody positivity gradually dropped to 50% by 6 months, the lowest level of 16% by 9 months, and rose to 34% by 12 months of age (**Table 2**). The age-wise distribution of neutralizing antibodies showed that 63%, 88%, 44%, 9%, and 22% of infants had multitypic responses at birth, 3, 6, 9, and 12 months, respectively. Nine infants had monotypic antibody profiles, and three infants were positive for DENV-1, -2, and -3 each (**Table 2**).

**Table 2:** Serotype-wise neutralizing and infection-enhancement antibodies in infants across different age groups.

	No. (%) of samples showing neutralizing activity					No. (%) of samples showing enhanced activity				
Age group (each n=32)	Any DENV	DENV-1	DENV-2	DENV-3	DENV-4	Any DENV	DENV-1	DENV-2	DENV-3	DENV-4
Cord blood	21 (66)*	20 (63)	21 (66)	19 (28)	18 (56)	21 (66)*	19 (28)	21 (66)	20 (63)	20 (63)
3 months	28 (88)**	24 (75)	27 (84)	28 (88)	27 (84)	28 (88)**^$^**	16 (50)	26 (81)	23 (72)	26 (81)
6 months	16 (50)***	13 (41)	14 (44)	14 (44)	11 (34)	27 (84)**^$$^**	18 (56)	23 (72)	22 (69)	22 (69)
9 months	5 (16)**^#^**	3 (9)	3 (9)	2 (6)	2 (6)	14 (44)**^@^**	3 (9)	11 (34)	10 (31)	8 (25)
12 months	11 (34)**^##^**	7 (22)	8 (25)	8 (25)	6 (19)	10 (31)	2 (6)	6 (19)	5 (16)	9 (28)

*all except one infant had monotypic neutralizing and enhancing activity against DENV-2; **all multitypic; ***14 multitypic, two monotypic (one each for DENV-1 and DENV-3); **^#^** three multitypic, two monotypic (one each for DENV-1 and DENV-2); **^##^** seven multitypic, four monotypic (one each for DENV-1 and DENV-2 and two for DENV-3); **^$^** 25 multitypic, three monotypic (two for DENV-2 and one for DENV-4); **^$$^** 22 multitypic, five monotypic (two each for DENV-2 and DENV-3 and one for DENV-4); **^@^** eight multitypic, six monotypic (three for DENV-2, two for DENV-3, and one for DENV-4); five multitypic, five monotypic (one for DENV-2 and four for DENV-4).

**Figure 1:**
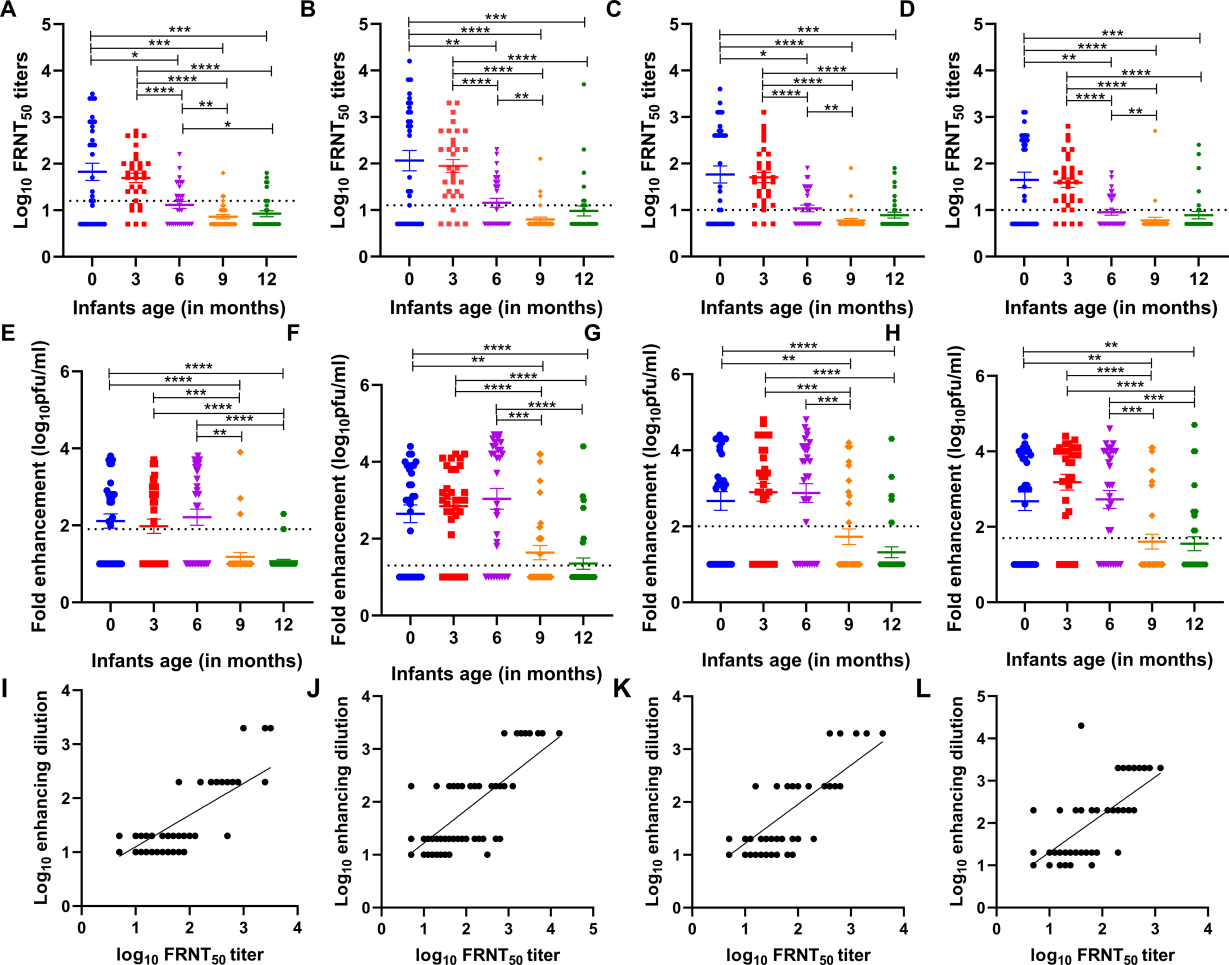
**(A-D)** Age-specific neutralizing antibody titers against each of the four dengue virus serotypes (DENV-1, DENV-2, DENV-3, and DENV-4) in the serum of n=32 infants each at birth i.e., 0, and 3, 6, 9, and 12 months of age. A baseline was drawn for each serotype which is the lower limit of quantitation (LLOQ), indicated by a dotted line (DENV-1 ≥ 16, DENV-2 ≥ 13, DENV-3 ≥ 11, and DENV-4 ≥ 11). P-values were calculated using the Mann–Whitney U-test (*p<0.01, **p<0.001, ***p<0.0001, ****p<0.0001). **(E-H)** Dengue virus serotype 1-4-specific positive infection enhancement at a particular dilution in serially diluted infant serum samples at different months of age (n=32 each at birth i.e., 0, and 3, 6, 9, and 12 months of age). Fold enhancement was measured as the fold increase in the DENV-infected cells relative to that in DENV-naive control. The dotted line indicates the cut-off value for each serotype above which positive enhancement was seen at different dilutions (DENV-1 ≥ 80, DENV-2 ≥ 20, DENV-3 ≥ 100, and DENV-4 ≥ 60 ffu/ml). P-values were calculated using the Mann–Whitney U-test (*p<0.01, **p<0.001). **(I-L)** Correlation of enhancing dilution (dilution at which enhancement of infection was observed) and neutralizing titers for all infant samples in a group including all 160 samples. A positive correlation was established by Spearman’s test (r=0.71, p<0.0001 for DENV-1; r=0.73, p<0.0001 for DENV-2; r=0.72, p<0.0001 for DENV-3; and r=0.74, p<0.0001 for DENV-4).

Higher geometric mean neutralizing antibody titers were observed at birth against all four DENV serotypes, with DENV-2 titers being the highest (**Figures 1A-D**). Comparable DENV1-4 neutralizing antibody titers were seen among infants at birth and 3 months of age. There was a significant drop in neutralizing antibody titers by 6 months compared to infants at birth (p=0.02 for DENV-1; p=0.01 for DENV-2, DENV-3, and DENV-4). At 6 months of age, the proportion of infants with detectable antibodies against DENV1-4 were 41%, 44%, 44%, and 34%, respectively. Furthermore, a significant decline in neutralizing antibody titers to the lowest levels was seen in 9-month-old infants as compared to 6-month-old infants (p=0.01, for DENV-1; p=0.001 for DENV-2 and DENV-3; p=0.003 for DENV-4). At 9 months of age, the proportion of infants with detectable antibodies against DENV1,-2,-3, and -4 were 9%, 9%, 6%, and 6%, respectively. The neutralizing antibody titers were comparable among 9 and 12-month-old infants (**Figures 1A-D**).

### 3.2 Reduced titers of maternally transferred DENV antibodies increase the likelihood of DENV infection enhancement in infants

Enhancing antibody activity was observed among 66%, 88%, and 84% of infants at birth, 3 months, and 6 months, respectively (**Table 2**). Subsequently, at 9 and 12 months of age, 44% and 31% of infants showed enhancing antibody activity, and ADE positivity was lowest in infants of 12 months of age. The age-wise distribution of enhancing antibodies showed that 63%, 78%, 69%, 25%, and 16% of infants exhibited enhancing antibody activity against more than one serotype at birth and at 3, 6, 9, and 12 months, respectively. Of 20 infants with monotypic enhancing antibody activity, 25% (5/20), 30% (6/20), and 25% (5/20) of infants were 6, 9, and 12 months of age, respectively (**Table 2**).

We observed comparable levels of antibody enhancement in infants of 3 months and 6 months of age and later declined significantly to lower levels at 9 and 12 months of age against all four serotypes (**Figures 1E-H**). Furthermore, we noted a significant positive correlation between enhancing dilution and FRNT_50_ titers across all four serotypes (r=0.71 to 0.74, p<0.0001 for all serotypes, **Figures 1I-L**).

**Figure 2:**
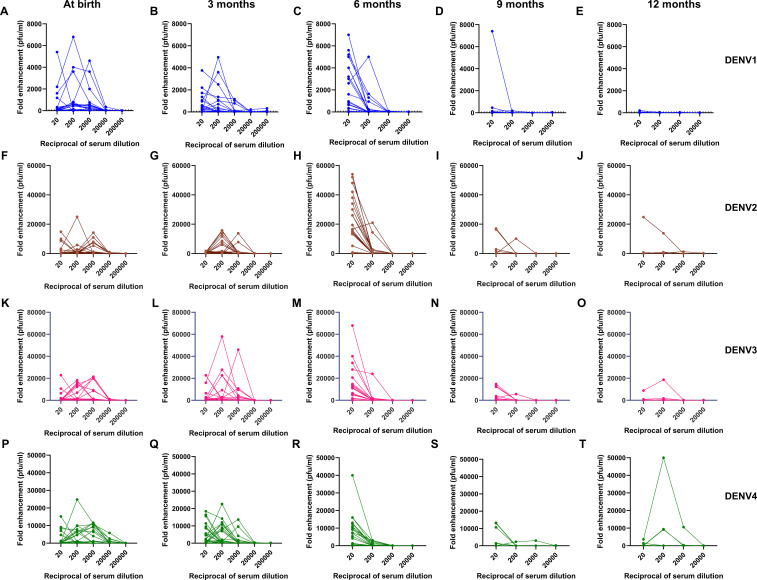
Kinetics of **(A-E)** DENV-1, **(F-J)** DENV-2, **(K-O)** DENV-3, and **(P-T)** DENV-4 infection enhancement in serially diluted infant serum samples at 0, 3, 6, 9, and 12 months of age (n=32).

**Figure 2** shows the enhancement of DENV-1, -2, -3, and -4 infection at different dilutions from 1:20 to 1:200,000, in infants across age groups. At birth, the maximum DENV infection was noticed at 1:200 dilution in 41% and 25% of infants for DENV-1 and DENV-3, respectively, whereas, for DENV-2 and DENV-4, the maximum enhancement was seen at 1:2000 dilution in 22% and 28% of infants, respectively. The median [IQR] range of enhanced DENV-1, -2, -3, and -4 virus infection was 50[1-580], 250[1-815], 410[1-2200], and 1270[30-5500], respectively, at 1:20 dilution when the sera of 3-month-old infants were examined. As the neutralizing antibody titers decreased, we noticed an increase in DENV-1, -2, -3, and -4 infection enhancement at 1:20 dilution with median [IQR] titers of 320[1-3,150], 9,200[5-26,000], 1,870[1-12,650], and 1,250[5-9,550], respectively, in 6-month-old infants. One-way ANOVA analysis was performed to assess DENV-1, -2, -3, and -4 infection enhancement at 1:20 dilution across different age groups (**Table 3**). Our data demonstrated infection enhancement of all four serotypes in 6-month-old infants in comparison to infants at birth, 9 months, and 12 months of age. Except for DENV-4, infection enhancement of the other three serotypes was also more prominent in 6-month-old than in 3-month-old infants (**Table 3**). This could probably be due to higher neutralizing antibody titers in infants until 3 months of age. Altogether, our data suggests that the highest fold enhancement was seen in infants of 6 months of age for all four serotypes at the lowest dilution of 1:20 (**Figure 2**).

**Table 3:** One-way ANOVA analysis of DENV-1, -2, -3, and -4 enhancing virus titers at 1:20 dilution of serum samples in infants across different age groups.

		DENV-1			DENV-2			DENV-3			DENV-4		
Sr. No.	Groups	Mean 1	Mean 2	p-value*	Mean 1	Mean 2	p-value*	Mean 1	Mean 2	p-value*	Mean 1	Mean 2	p-value*
1	at birth vs. 3m	381	449	0.9995	1274	438	0.9947	1479	2699	0.9698	1565	3794	0.3773
2	at birth vs. 6m	**381**	**1539**	**0.0028**	**1274**	**14536**	**<0.0001**	**1479**	**9239**	**0.0009**	**1565**	**5518**	**0.0145**
3	at birth vs. 9m	381	260	0.9953	1274	1210	>0.9999	1479	1566	>0.9999	1565	1290	0.9995
4	at birth vs. 12m	381	11	0.7636	1274	805	0.9994	1479	328	0.9755	1565	199	0.8048
5	3m vs. 6m	**449**	**1539**	**0.0058**	**438**	**14536**	**<0.0001**	**2699**	**9239**	**0.0079**	3794	5518	0.6333
6	3m vs. 9m	449	260	0.9745	438	1210	0.9961	2699	1566	0.9769	3794	1290	0.2605
7	3m vs. 12m	449	11	0.6308	438	805	0.9998	2699	328	0.7354	**3794**	**199**	**0.0339**
8	6m vs. 9m	**1539**	**260**	**0.0007**	**14536**	**1210**	**<0.0001**	**9239**	**1566**	**0.001**	**5518**	**1290**	**0.0072**
9	6m vs. 12m	**1539**	**11**	**<0.0001**	**14536**	**805**	**<0.0001**	**9239**	**328**	**<0.0001**	**5518**	**199**	**0.0003**
10	9m vs. 12m	260	11	0.9321	1210	805	0.9997	1566	328	0.968	1290	199	0.9035

Mean 1 and Mean 2 represent enhancing antibody titers of the two groups being compared. *significant p-values are highlighted in bold.

## 4 Discussion

This is the first comprehensive study from India illustrating the decline in maternally acquired dengue-neutralizing antibodies concordant with the enhancement of dengue infection among healthy infants by 6 months of age. We established a direct association between the enhancing dilution and FRNT_50_ titers (**Figures 1I-L**). The higher the antibody levels, the greater the dilution to reach sub-neutralizing levels at which Fc-gamma-associated ADE occurs.

In dengue-endemic countries, most newborn infants exhibit high levels of dengue antibodies at birth as most women of child-bearing age are immune to all four DENV serotypes. Our previous study also showed high endemicity of dengue in Pune with more than 80% of anti-dengue IgG seropositivity in adults (Mishra et al., 2018). Multiple studies conducted in Bangkok, Thailand, between 1998 and 2001 showed that more than 80% of infants lost maternal DENV-neutralizing antibodies by 9 and 12 months of age (Pengsaa et al., 2006; Watanaveeradej et al., 2003; Pengsaa et al., 2003). Here, we report that 50% of Indian infants lost maternally acquired DENV-neutralizing antibodies by 6 months, and by 9 months of age, maternally acquired neutralizing antibodies disappeared in 84% of infants. Our findings are in line with a study of healthy Thai infants where 80% of infants were devoid of neutralizing antibodies by 9 months of age (Pengsaa et al., 2011). In healthy Vietnamese infants, almost all infants lost the neutralizing antibodies by 6 months of age (Chau et al., 2009). In contrast, maternally acquired DENV-3-specific neutralizing antibodies disappeared in >90% of infants by the age of 4 months in a Brazilian cohort (Castanha et al., 2016). Clearly, the decay of neutralizing antibodies is faster in Brazilian infants than in Asian infants.

Another important factor is the role of the maternal antibodies transferred through breast milk in protection/enhancement that could differ among breastfed and formula-fed infants. Breastfeeding usually facilitates the transfer of IgA, IgM, and IgG antibodies (Lokossou et al., 2022). Secretory IgA is found to be higher in breast-fed infants than the formula-fed infants (Avanzini et al., 1992). An early report revealed that anti-dengue neutralizing activity in human milk did not decrease for 10 months after delivery. After isolating the immunoglobulins, no anti-dengue activity was observed (Falkler et al., 1975). In another study, DENV1-specific maternal IgG antibodies were acquired from breastfeeding in a maternal antibody transfer mouse model (Lee et al., 2016). In the absence of data on the type and duration of feed type, we cannot comment on the impact of feed type on the results obtained in this study.

While many clinical studies have suggested an indirect link between declining maternal antibodies and severe dengue, hypothesizing ADE of infection, only two studies measured and confirmed this infection enhancement to a single serotype (Chau et al., 2008; Castanha et al., 2016). We confirm the findings of a study from Vietnam on dengue serotype 2 (Chau et al., 2008) and extend them to all four serotypes, demonstrating peak antibody enhancement by the age of 6 months. Unlike Asian infants, Brazilian infants born to DENV-3 immune mothers experience DENV-2 infection enhancement by 2 months of age and rapidly decline by the age of 4 months (Castanha et al., 2016). Notably, when hospitalized Vietnamese infants were evaluated for antibody response, 65% of infants experienced dengue hemorrhagic fever when the maternally acquired neutralizing antibody titer had declined to <1:20 against their infecting serotype (Simmons et al., 2007). Our results suggest that by 6 months of age, as the neutralizing antibody levels against DENV rapidly decreased, 84% of infants maintained ADE activity to at least one serotype at the lowest serum dilution of 1:20. Another study by Libraty et al. found that almost all Filipino infants with symptomatic DENV-3 infections had sub-neutralizing plasma IgG levels and measurable Fc receptor-dependent DENV-3 ADE activity at the time of infection (Libraty et al., 2009).

Halstead first reported that infants experiencing their first exposure to primary dengue often present with severe forms of the disease and require hospitalization in Southeast Asia (Halstead et al., 2002). Similar investigations from India documented severe dengue in children and in nearly all infants under 1 year old, further reinforcing these findings (Aggarwal et al., 2024; Dash et al., 2021; Kabilan et al., 2003). A recent study from India indicated that 65% of infants younger than 1 year had severe diseases mainly attributed to primary infections (Aggarwal et al., 2024). A retrospective evaluation of 10 years of medical records from between 2009 and 2019 revealed that almost half of the admitted infants had severe dengue (Dash et al., 2021). Data from outbreak investigations in the 2001 dengue epidemic in Chennai revealed that 20% of infants required hospitalization with no mortality (Kabilan et al., 2003). Supporting this, recent studies have linked age-specific declining antibody patterns to infant dengue hospitalizations, emphasizing the relative risk of severe dengue among infants (O’Driscoll et al., 2023; Clapham et al., 2015). Overall, these findings support mathematical model predictions that the critical time for severe dengue occurrence is roughly 2 months after maternal dengue-neutralizing antibodies degrade below a protective level (Adimy et al., 2020).

A limitation of our study is the retrospective cross-sectional nature which lacks follow-up samples from the same infant. However, considering the study on Vietnamese infants that used both cross-sectional and longitudinal sets of samples and showed similar patterns of antibody levels and infection enhancement (Chau et al., 2008), our results will most likely be similar when follow-up samples are used.

In conclusion, our study highlights that maternally transferred neutralizing antibodies were undetectable in 84% of infants by 9 months of age. The highest level of DENV infection enhancement across all four serotypes was documented in 6-month-old infants at the lowest reciprocal dilution of 1:20. Our study suggests that maternally acquired neutralizing antibodies could be protective until 6 months of age and capable of facilitating antibody-dependent enhancement on exposure to dengue infections in later months of life.

## Data Availability

The original contributions presented in the study are included in the article/supplementary material. Further inquiries can be directed to the corresponding author.
